# Game controller modification for fMRI hyperscanning experiments in a cooperative virtual reality environment

**DOI:** 10.1016/j.mex.2014.10.009

**Published:** 2014-11-04

**Authors:** Jason Trees, Joseph Snider, Maryam Falahpour, Nick Guo, Kun Lu, Douglas C. Johnson, Howard Poizner, Thomas T. Liu

**Affiliations:** a Institute for Neural Computation, University of California San Diego, 9500 Gilman Drive - 0523, La Jolla, CA 92093-0523, United States; b Center for Functional MRI, W. M. Keck Building, University of California San Diego, 9500 Gilman Drive – 0677, La Jolla, CA 92093-0677, United States; c Warfighter Performance Department, Naval Health Research Center, 140 Sylvester Rd, San Diego, CA 92106-3521, United States; d Department of Psychiatry, University of California San Diego, 9500 Gilman Drive - 0603, La Jolla, CA 92037-0603, United States

**Keywords:** Hyperscanning, fMRI, MRI compatible, Game controller, Virtual environment, Cooperative simulation

## Abstract

Hyperscanning, an emerging technique in which data from multiple interacting subjects’ brains are simultaneously recorded, has become an increasingly popular way to address complex topics, such as “theory of mind.” However, most previous fMRI hyperscanning experiments have been limited to abstract social interactions (e.g. phone conversations). Our new method utilizes a virtual reality (VR) environment used for military training, Virtual Battlespace 2 (VBS2), to create realistic avatar-avatar interactions and cooperative tasks. To control the virtual avatar, subjects use a MRI compatible Playstation 3 game controller, modified by removing all extraneous metal components and replacing any necessary ones with 3D printed plastic models. Control of both scanners’ operation is initiated by a VBS2 plugin to sync scanner time to the known time within the VR environment. Our modifications include:

• Modification of game controller to be MRI compatible.
• Design of VBS2 virtual environment for cooperative interactions.
• Syncing two MRI machines for simultaneous recording.

## Graphical abstract

**Figure:**
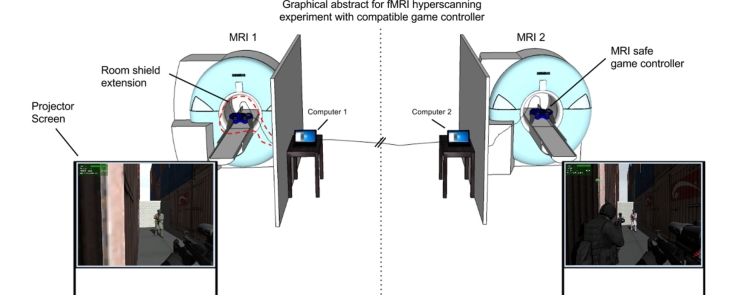

The Magnetic Resonance Imaging (MRI) environment presents unique challenges to the introduction of any additional hardware into close proximity with the machine. If proper care and safety are not taken when bringing equipment into the bore, the strong magnetic field creates conditions that could harm subjects or the machine. In addition, inclusion of any active electronic device must be tested to make sure there is no interference with data collection or induction of artifacts into the data. Devices, like a computer keyboard, have previously been used to record complex motor responses within an MRI and have been determined to be both safe and to not interfere with data quality [1]. However, for natural, immersive control of a virtual avatar, a game controller with dual analog joysticks has been shown to be more effective and has become the commercial game industry standard. A modified game controller has been used within an MRI to control action of a single agent within a complex virtual representation (e.g. the path of a virtual taxi cab in London [2]), but details on the necessary modifications have not been previously published. Recording simultaneously from two separate brains has been shown to elucidate neural markers of subject–subject interaction, but has been mainly limited to social experiments [3]. Through the use of virtual avatar representations controlled simultaneously by the two subjects, the ambiguity of cooperative interaction is kept to a minimum leaving only the salient time series and events for analysis. The following is a detailed procedure for modifying a game controller to be MRI compatible, along with steps for creating hyperscanning virtual environments. As MRI-compatible controllers with the functionality required for hyperscanning in a virtual environment are not currently available on a commercial basis, the procedures described should prove to be useful for the development of experimental protocols in this area of research.

## Method details

### Step 1: Game controller modification

#### Materials

• Snakebyte^®^ basic wired controller (SB00566) v1.1 (Purchased from Walmart)
• Belden 8723 060 (CHR) 1000 Ft 305 M, Special purpose audio communication & instrumentation cable -2 pair, Each pair Beldfoil^®^ SHIELDED (Purchased from http://www.mouser.com)
• D-Sub 25 pin male and female connector kits (or similar connector) (Purchased from http://www.mouser.com)3D Systems ProJet^®^ 3510 HD
• Super Shield™ Silver Coated Copper Conductive Coating (MG Chemicals) (Purchased from http://www.mouser.com)
• McMaster-Carr 3/8″ Slotted Brass Round Headed Screws (92407A079) (Purchased from www.mcmaster.com)
• McMaster-Carr Ultra-precision 302 Stainless Steel Compression Spring (9002T15) (Purchased from http://www.mcmaster.com)
• Loctite^®^ 416 Super Bonder Instant Adhesive (Part No. 41650) (Purchased from Walmart)

Note: This list includes only non-standard materials. Standard equipment such as a soldering iron, wire-cutters, etc., are assumed to be available.

#### Procedure

**Fig. 1:**
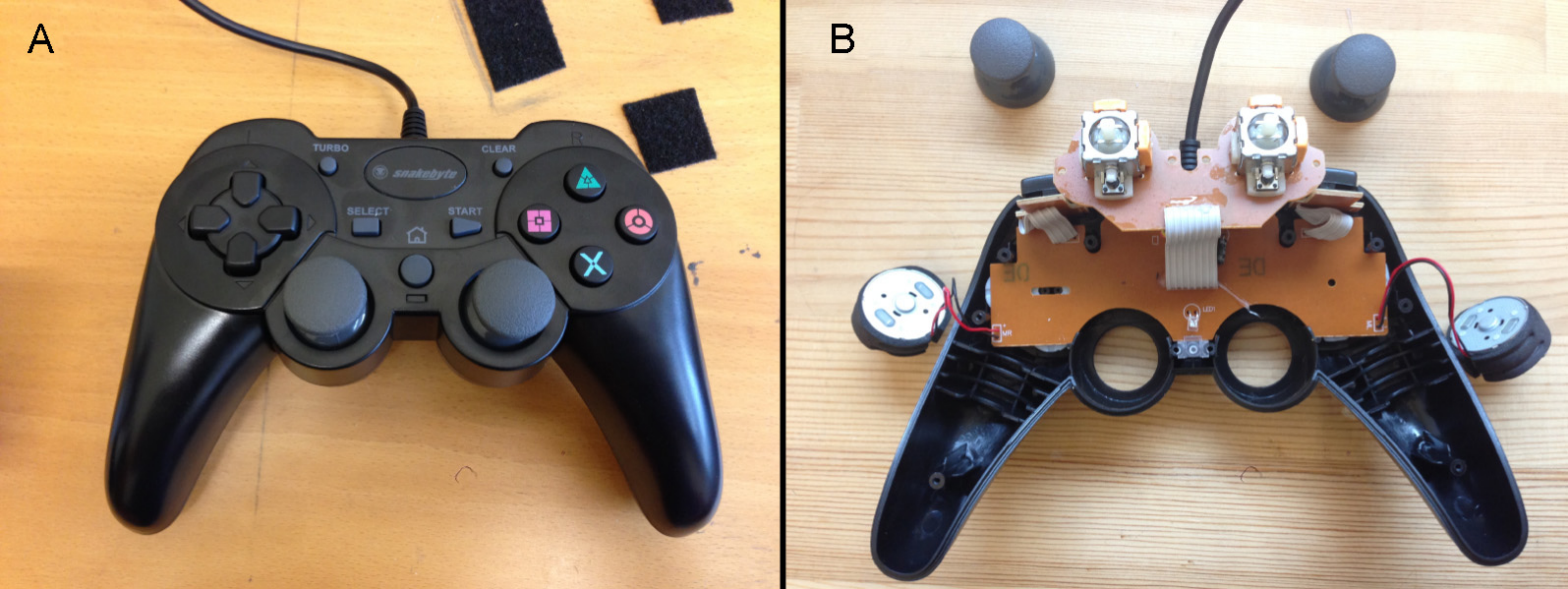
(A) Snakebyte^®^ basic wired controller. (B) Reverse view with back casing removed and circuit board folded out for viewing.

**Fig. 2:**
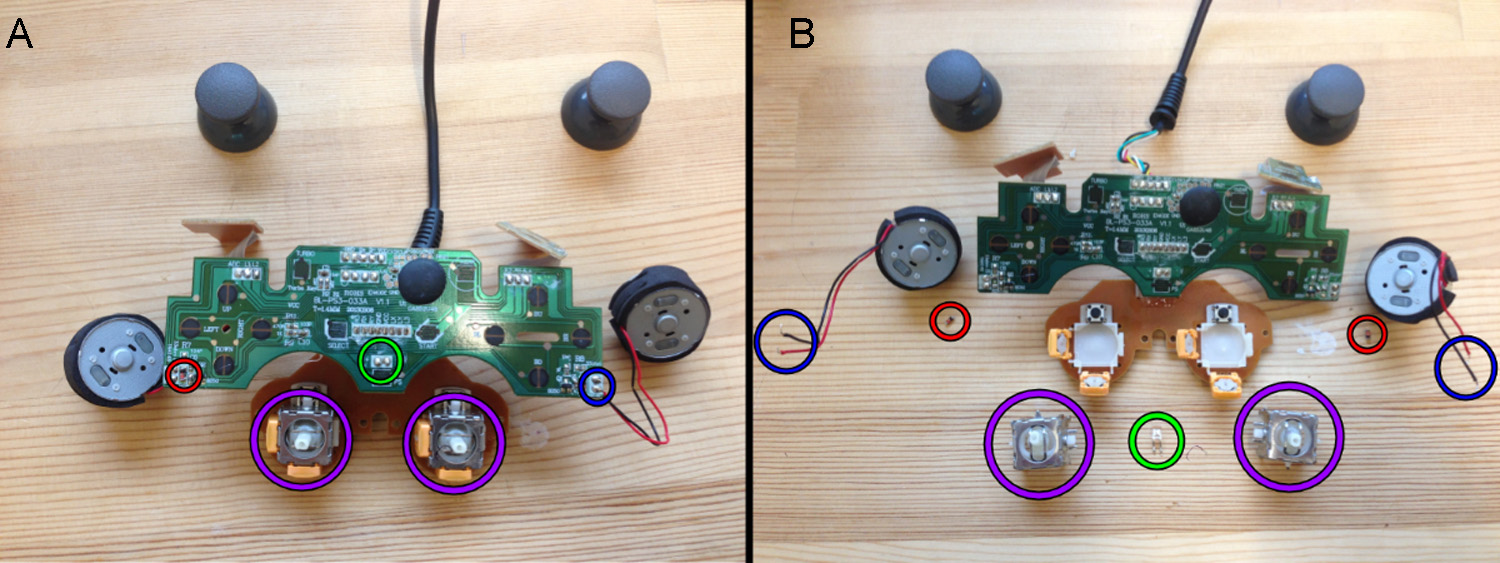
(A) Controller circuit board with highlighted parts to be removed. (B) Controller circuit board after previously highlighted corresponding parts were removed.

**Fig. 3:**
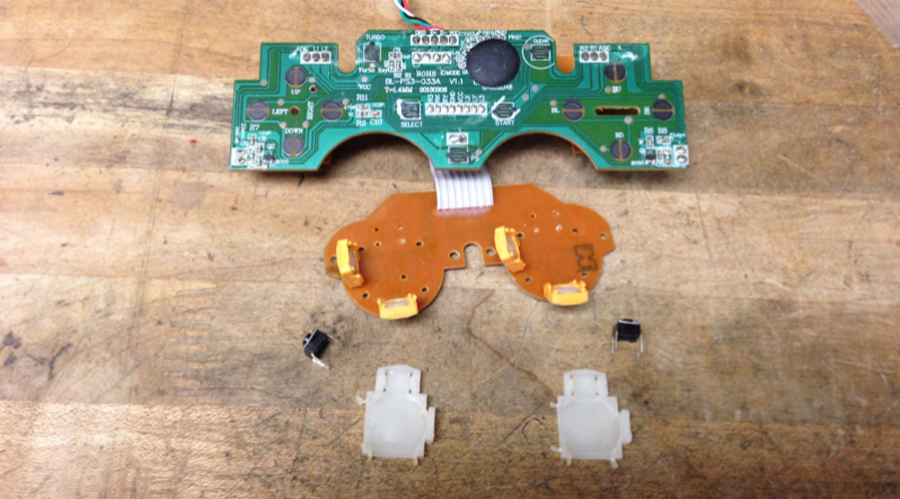
Circuit board with joystick base (white) and button (black) completely removed.

**Fig. 4:**
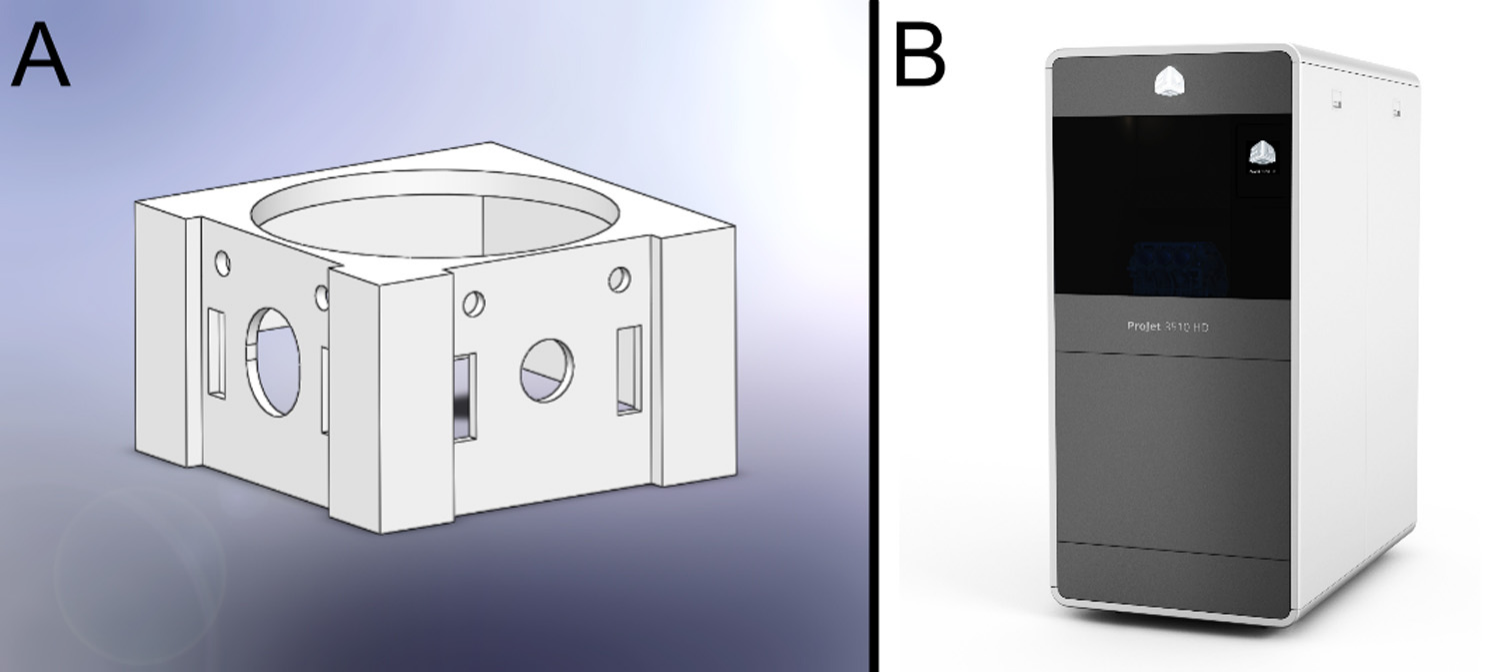
(A) SolidWorks 2013 generated 3D model of joystick housing. (B) 3D Systems ProJet^®^ 3510 HD printer.

**Fig. 5:**
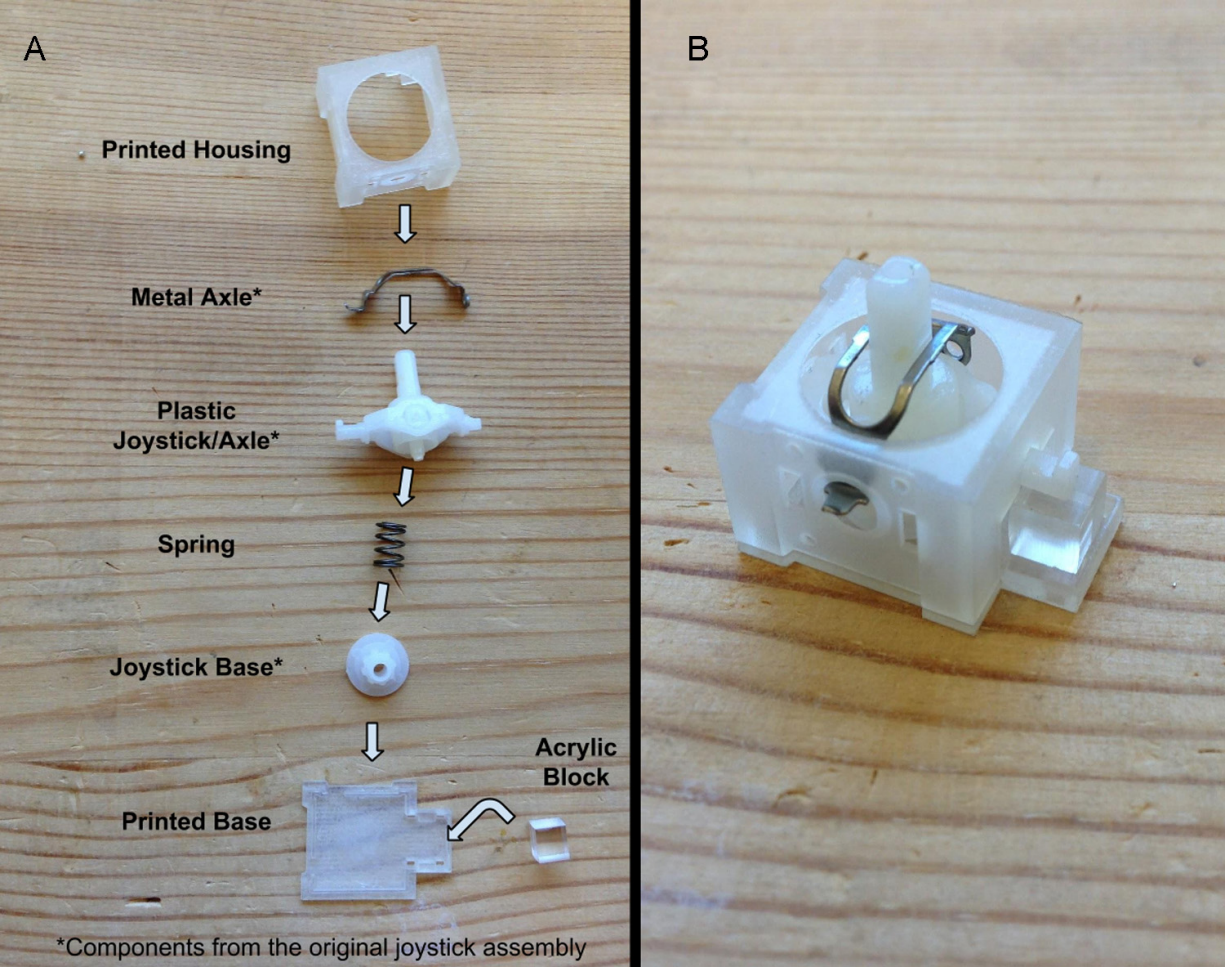
(A) A step-by-step layout of the pieces necessary to reconstruct the joystick assembly. The component names with an asterisk are from the stock joystick assembly and do not need to be made. (B) Fully re-assemble, MRI safe, joystick assembly.

**Fig. 6:**
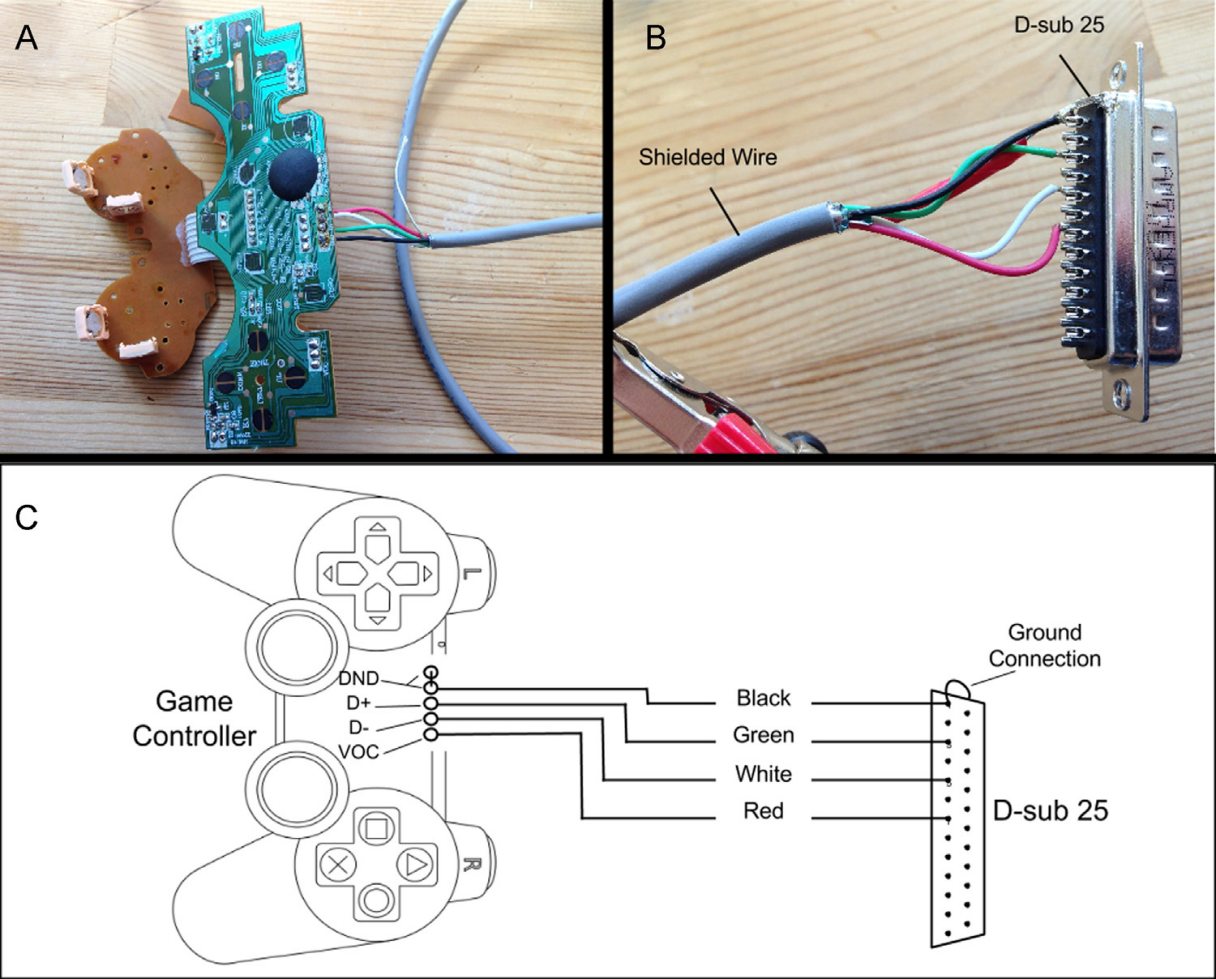
(A) Circuit board with new shielded wire cable connected. (B) Corresponding D-Sub 25 connector attachment on the other end of the shielded cable. (C) Wiring Diagram detailing the specific connections and wires used.

**Fig. 7:**
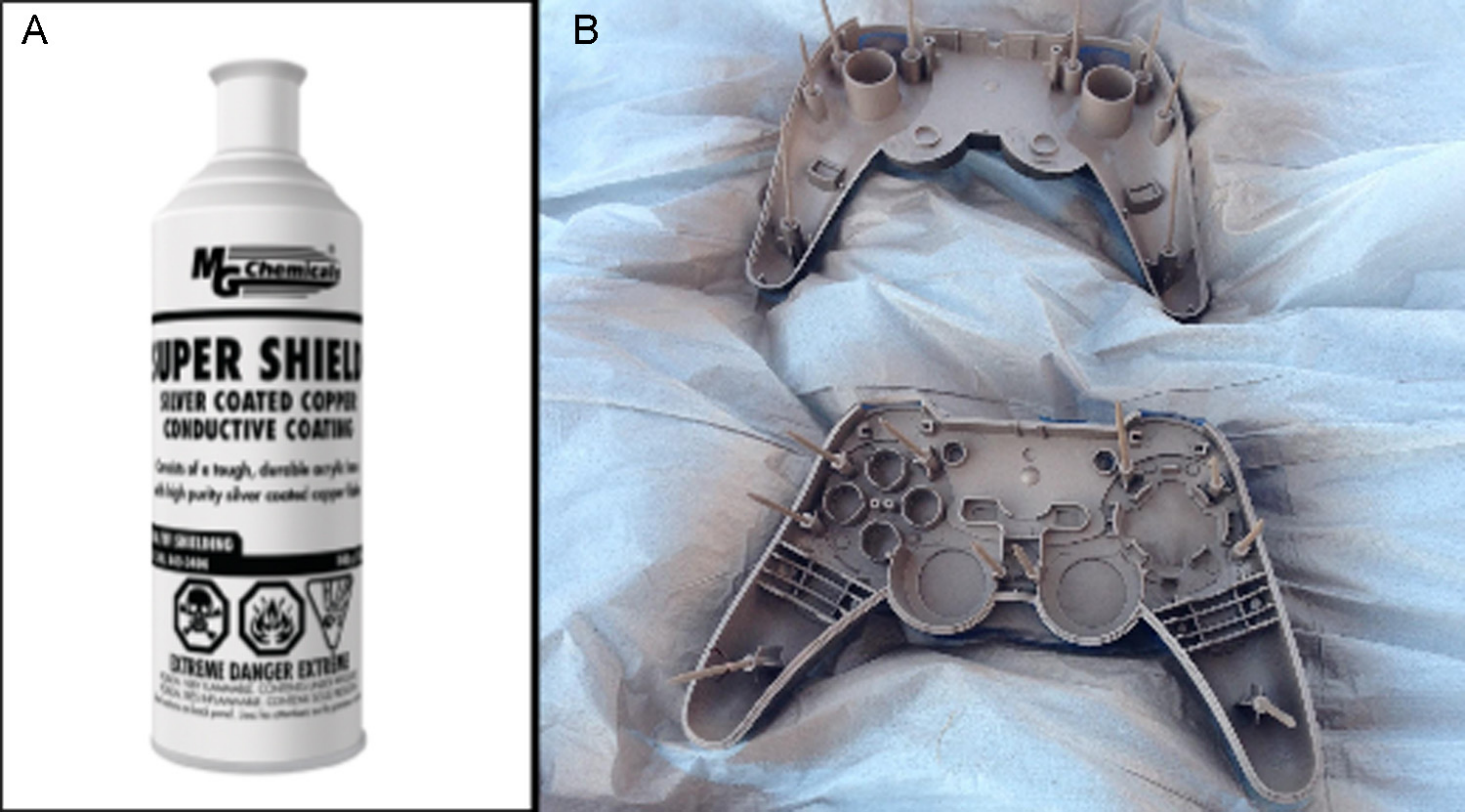
(A) Super Shield™ Silver Coated Copper Conductive Coating spray can. (B) Controller casings with the inside coated with shielding paint. Toothpicks were placed in the screw holes to prevent closure prior to painting.

**Fig. 8:**
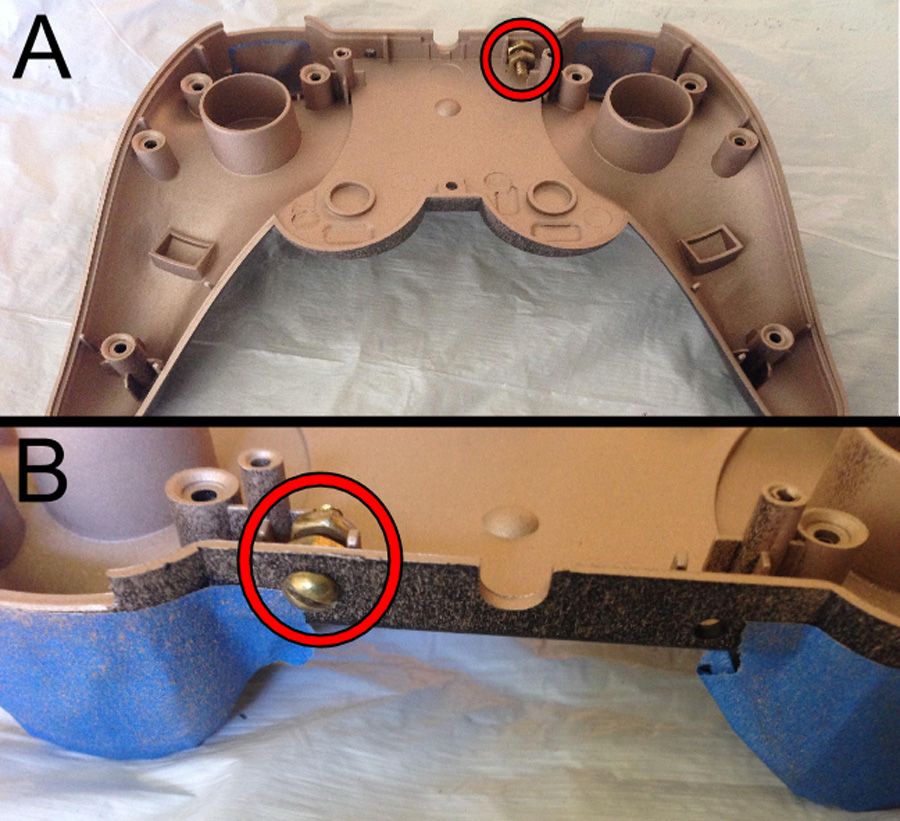
(A) Front view of the brass nut and bolt placement used to secure shielding wire. (B) Reverse view of bolt placement.

1. Remove the controller back and fold out the smaller circuit board (Fig. 1)
2. Completely remove the circuit board from the controller housing. Keep all of the components to the buttons and plastic joystick covers.
3. With a soldering iron remove the following components (Fig. 2):
  a. Two pairs of wires leading to the vibrator motors (circled in blue)
  b. Two adjacent resistors for vibrator function (circled in red)
  c. Two joystick assemblies (circled in purple)
    i. Note: bend back the orange potentiometers carefully so as to not break any of the connections with the circuit board
  d. One LED (circled in green)
4. Remove the white plastic base of the joystick assembly along with the attached button (Fig. 3).
5. Rebuild the joystick assembly:
  a. Using SolidWorks 2013, create a 3D computer model of the metal joystick housing and plastic base (Fig. 4A).
  b. Use 3D Systems ProJet^®^ 3510 HD printer (Fig. 4B) to print a precise replication of the computer generated model with VisiJet M3 Materials (or other MRI safe plastic like ABS or PLA).
  c. Disassemble the stock joystick assemblies, keeping the plastic components and the single (non-magnetic) metal axle.
  d. Cut the ultra-precision 302 stainless steel compression spring to the same size as the stock component
  e. Glue a plastic block, cut to the dimensions of the joystick button, to the base piece in lieu of the button (Fig. 5A). This supports one of the joystick axles. Note that the button is not needed to control the virtual environment. It is replaced in the present implementation due to the presence of magnetic metal within the button.
  f. Reassemble the joystick using the printed housing and base, along with ultra-precision 302 stainless steel compression spring and remaining stock pieces (Fig. 5A).
  g. Secure the assembly by gluing the housing and base together on the outside, making sure to not allow any glue to reach the inside (Fig. 5B).
6. Attach the rebuilt joystick assembly to the circuit board and secure the potentiometers to the new housing.
  a. Using the Unity Web Player – joystick test web application (see http://dal-acm.ca/∼dice/joytest/joystick%20test.html), test to make sure the buttons and potentiometers for the joysticks are responding properly.
7. Assemble new controller wire and connector:
  a. Using a soldering iron remove the stock USB wire connections.
  b. Cut 20 feet (6.096 meters) of Belden 8723 Special purpose audio communication & instrumentation cable-2 pair.
  c. At both ends, strip and separate the wires.
  d. For the first twisted pair, solder one wire to the connection marked D- and one to the connection marked D+.
  e. For the second twisted pair, solder one connection to the ground, DND, and one to the power, VOC.
  f. On the other side of the cable, solder the wires to a D-sub 25 connector, using pins 1, 3, 5, and 7, for the wire leading to DND, D+, D-, and VOC, respectively (Fig. 6). Any connector with 4 pins or more can be used to the same effect (e.g. 2 BNC or serial port).
  g. Solder a further connection from the ground pin (1), to the metal housing of the D-sub 25 connector. This will effectively connect the room shielding to the controller shielding. No external filter is used.
8. Build secondary D-sub 25 to USB wire.
  a. Make sure to match the correct USB wire configuration with the correct pins used in step 7. This is to connect the MRI room wall pass-through to the experiment computer.
9. Apply shielding to controller housing.
  a. Drill a hole in bottom half of the casing near the cable opening.
  b. Using painter's tape, tape off all holes and the sides of each housing piece on the outer side.
  c. Insert toothpicks into the screw holes to prevent blockage from paint application.
  d. Spray an even and solid coat of Super Shield™ Silver Coated Copper Conductive Coating (Fig. 7A) on the inside of both halves (Fig. 7B).
    i. Make sure to apply the paint to the edges of each half where they make contact with each other when reassembled.
  e. Wait 15 min and spray a second coat.
  f. Wait 24 h and repeat steps c and d.
  g. Allow an additional 24 h to cure after final coat.
10. Insert a brass bolt into the drilled hole and secure tightly with a brass nut (circled in red). Attach a second brass nut without fully tightening it (Fig. 8).
11. Replace all of the button pieces into the controller housing.
12. Insert the circuit board back into the housing.
13. Secure the circuit board with the replacement 3/8″ slotted brass round headed screws.
14. Attach the “naked” shielding wire from the cable to the brass bolt in controller housing and secure with the second nut.
15. Reconnect the two halves of the controller housing and secure with brass screws.

### Step 2: Virtual environment construction

#### Materials

• Virtual Battlespace 2 (VBS2, Bohemia Interactive Simulations, available from http://www.bisimulations.com/)
  ∘ Minimum System requirements (Obtained from ARMA II a commercial game that uses the VBS2 engine http://www.arma2.com):
    • CPU: Dual Core Intel Pentium 4 3.0 GHz/Intel Core 2.0 GHz/AMD Athlon 3200+ or faster
    • CPU Speed: Dual Core Intel Pentium 4 3.0 GHz/Intel Core 2.0 GHz/AMD Athlon 3200+ or faster
    • RAM: 1 GB
    • OS: Windows XP or Vista
    • Video Card: NVIDIA GeForce 7800/ATI Radeon X1800 with Shader Model 3 and 256 MB VRAM or faster
• Two VBS2 HASP dongle licenses

#### Procedure

**Fig. 9:**
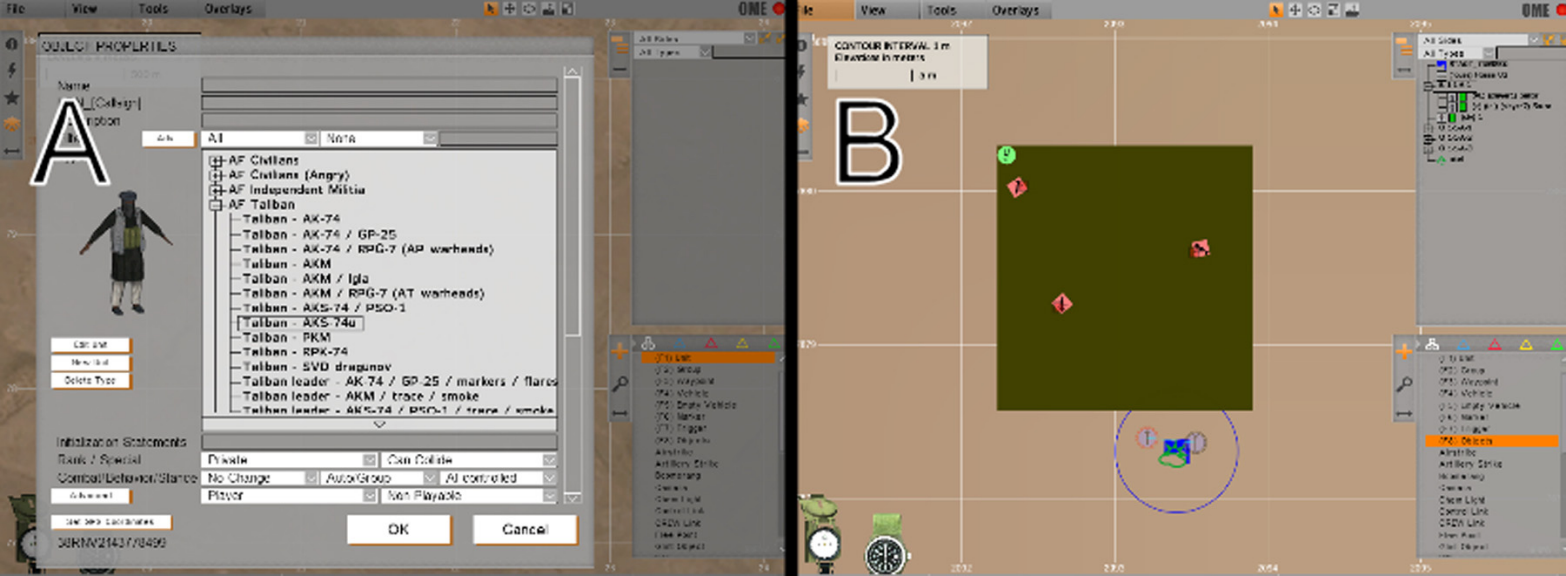
(A) VBS2 character and avatar selection interface. (B) In-game event placement and programming interface.

1. Obtain objects and actors, along with a suitable environment for the scenario, using VBS2's development interface and object libraries (Fig. 9A).
2. Set situational logic and player/AI settings via VBS2's scripting language to streamline the development process and provide extensibility (Fig. 9B).
  a. Establish this by creating an initialization script that triggers when the scenario first starts along with other script files that contain particular functions.
  b. All scripts should all be placed in the same folder as the mission's files and have the file extension .sqf (See Supplementary Material below)
3. Bind the controller keys to basic in-game functionality by assigning appropriate actions to each of the keys through the options menu.
4. Create non-verbal communication interface
  a. Develop separate functions using VBS2's scripting language to display text locked to avatar position
  b. Assign the actions to the “User Defined Actions” option in the script.
  c. Bind the various “User Defined Actions” to the appropriate controller buttons using the options menu.
5. Set-up additional data logging.
  a. Locate the correct scripting functions to interact with VBS2's native logger, After Action Review (AAR).
  b. Within the initialization script, add additional events to be logged into the AAR such as reaching a checkpoint, firing shots, and signaling to each other.
6. Initiate cooperative mission over Local Area Network (LAN) using VBS2's built-in networking protocol.

### Step 3: Dual MRI scanner control

#### Materials

• Local Area Network (LAN) switch
• Ethernet cables
• USB Teensy 3.1, PJRC, LLC, http://www.pjrc.com/teensy
• BNC connector and cable

#### Procedure

1. Solder the BNC connector to the 12-bit DAC channel (D) and ground (G) on the Teensy 3.1.
2. Install Arduino software for flashing the Teensy 3.1 (http://www.arduino.cc/en/Main/Software)
  a. Open the included link above.
  b. Select the download link for the user's operating system.
  c. Launch installer and follow the prompts.
3. Install the Teensy extension to the Arduino Library (https://www.pjrc.com/teensy/td_download.html)
  a. Open the included link above.
  b. Select the download link for the user's operating system.
  c. Launch installer and follow the prompts.
4. Connect the Teensy 3.1 to the computer with its mini USB cable.
  a. Only one Teensy is needed to trigger the two scanners from the master experiment computer.
5. Flash the Teensy 3.1 with custom code with:
  a. Serial interface to receive commands
  b. Ability to send a 10 ms square wave pulse.
6. A custom plugin for VBS2 allows it to connect to the Teensy 3.1 over a serial connection and send a pulse over BNC to both MRIs.
7. Initializing the two MRIs simultaneously allows two consecutive pulses (500 ms apart) to start both MRIs in sync from within the VBS2 environment.

## Additional Information

### Noise Tests

In addition to modifying the game controller to be safe for use within a MRI by removing all magnetic components, tests to determine whether or not the device will introduce excessive noise or artifacts into the MRI data were conducted. The scanners employed are the GE Discovery MR750 3T. Each scanner was used in conjunction with an in vivo 8 channel head coil. The tests were run with the controller plugged in, turned on, and placed within the bore at the approximate location it would be during an experiment. In addition, buttons were continuously pressed throughout the test by an experimenter located just outside the bore to simulate the hardware in use. The Center for Functional MRI at UC San Diego School of Medicine has established a signal to fluctuation noise ratio (SFNR) reading of 350 as the cut-off for an acceptable level of stability (see http://www.birncommunity.org/tools-catalog/function-birn-stability-phantom-qa-procedures/ for description). In addition, values for RMS stability and Weisskoff stability (RDC) [4] are obtained to give a more comprehensive analysis of scanner stability. A baseline reading without a controller was obtained, labeled phantom in Table 1, and had a SFNR of 388.3. Both controllers were run in the same noise test protocol in the conditions described above and resulted in SFNR readings of 375.0 and 377.7, respectively. Both of these values are well above the SFNR cut-off of 350, indicating a minimal level of noise induction by the modified game controller.

Along with testing the effect of the controller on fMRI data collection, the controllers were tested for induction of artifacts by the MRI on controller functionality. Both controllers displayed no artifacts in functionality for the entirety of the 10 min scans (data not shown).

## Figures and Tables

**Table 1:** Controller noise tests.

Controller	RMS stability	SFNR	Weisskoff stability (RDC)
Controller 1^a^	0.037	375.0	7.4
Controller 2^a^	0.031	377.7	9.4
Phantom	0.030	388.3	9.5

a Both controller 1 and controller 2 passed the stability test requirements (SFNR > 350; RMS Stability < 0.06; RDC > 4.5) used for the system quality assurance process at the UCSD Center for Functional MRI.
