# Supplementary material for: Game controller modification for fMRI hyperscanning experiments in a cooperative virtual reality environment
Source: MethodsX. 2014 Nov 4;1:292–9. doi: 10.1016/j.mex.2014.10.009 (PMC4473038; doi:10.1016/j.mex.2014.10.009)
Supplement: Supplementary file 2 [file mmc2.pdf]

Supplementary Material

init_script.sqf:
Initializes the units in the scenario with different settings depending on whether or not they are players and binds signaling functionality for communication so that they can be executed via controller input. Also, adds event handlers for logging purposes and finally executes the plugin, which sends out the TTL pulse that starts both MRI machines synchronously. NOTE: for the signaling to work, "UserDefActionX" must be mapped accordingly to a controller button in the VBS2 control panel.

save_hostage.sqf:
Ends the mission with a success. Can customize this file for more functionality to be executed before the mission completes.

fullRespawn.sqf:
Respawns all units and players to allow for extended scenarios.

mission files:
Scenario files created via the VBS2 interactive scenario development environment. Only has to run on the master machine since the slaves connect and download these files.

VBSTeensyTTL:
A Microsoft Visual Studio 2012 (www.visualstudio.com) project for building the plugin to VBS2. It requires VBS2 (www.bisimulations.com) and BOOST (www.boost.org) to be installed.

serialTeensy_ino.ino:
TeensyDuino code (www.pjrc.com/teensy) to set up the Teensy 3.1 to pulse a TTL signal on command via a serial port.
